# Localisation of the petrous internal carotid artery relative to the vidian canal on computed tomography: a case–control study evaluating the impact of petroclival chondrosarcoma

**DOI:** 10.1007/s00701-022-05254-2

**Published:** 2022-05-25

**Authors:** Steve E. J. Connor, Nicholas W. M. Thomas, Jonathan Shapey

**Affiliations:** 1 School of Biomedical Engineering and Imaging Sciences, St Thomas’ Hospital, King’s College, London, SE1 7EH UK; 2 Department of Neuroradiology, King’s College Hospital, Denmark Hill, London, SE5 9RS UK; 3 Department of Neurosurgery, King’s College Hospital, London, SE5 9RS UK

**Keywords:** Base of skull, Chondrosarcoma, Petrous apex, Carotid artery, Internal, Angiography, Computed tomography, Cross-sectional anatomy

## Abstract

**Background:**

The vidian canal (VC) is normally a reliable anatomical landmark for locating the petrous internal carotid artery (pICA). This study determined the influence of petroclival chondrosarcoma on the relationship between the VC and pICA.

**Methods:**

Nine patients (3 males, 6 females; median age 49) with petroclival chondrosarcoma, and depiction of the pICA on contrast-enhanced CT, were retrospectively studied. CT-based measurements were performed by two observers, both in the presence of the petroclival chondrosarcoma (case) and on the contralateral control side. The antero-posterior (AP) and craniocaudal (CC) measurements from the posterior VC to the pICA, whether the pICA was in the trajectory of the VC, and the coronal relationship of the pICA anterior genu with the VC were recorded.

**Results:**

Chondrosarcoma usually displaced the pICA anteriorly (8/9 cases) and superiorly (6/9 cases) relative to the normal side with mean AP and CC measurements of 3.9 mm v 7.2 mm (*p* = 0.054) and 4.4 mm v 1.4 mm (*p* = 0.061). The VC trajectory less frequently intersected the pICA cross-section in the presence of chondrosarcoma however it was in the line of the eroded dorsal VC in one case. The anterior genu of the pICA was displaced more laterally by chondrosarcoma but usually remained superior to the VC.

**Conclusion:**

Petroclival chondrosarcoma variably influences the anatomical relationship between the VC and the pICA, hence requiring an individualised approach. The pICA is usually anterosuperiorly displaced, and the anterior genu remains superior to the VC, however it may be located in the line of the canal.

## Introduction

Endoscopic endonasal approaches (EEA) offer a direct approach to the resection of petroclival lesions, without the need for brain retraction [9, 17, 25]. The surgical corridor requires the localisation and exposure of the petrous internal carotid artery (pICA) in order to safely remove tumour [13]. The relationship of anatomical landmarks with the adjacent pICA has been evaluated in studies of cadaveric, dry skull and histological specimens [3, 5, 7, 8, 12, 14, 15, 18, 19, 21, 22] as well as on computed tomography (CT) imaging [12, 15, 16, 23].

The vidian canal (VC) is widely accepted as being a reliable indicator of the pICA location in the coronal plane [3, 5, 7, 8, 12, 14, 15, 21, 23]. In normal subjects, the pICA courses anteromedially to the foramen lacerum where it curves superiorly at the anterior genu [5] to become the paraclival ICA. The VC runs posterolaterally from the pterygopalatine fossa to reach the anterolateral margin of the foramen lacerum where its posterior opening usually lies inferior to the pICA and inferolateral to its anterior genu [8, 12–14, 21, 23].

Chondrosarcomas of the skull base are indolent locally invasive neoplasms which represent 6% of all skull base tumours [4, 6] and typically originate from the cartilaginous elements of the petroclival synchondrosis [2]. Many centres advocate EEA as the initial surgical approach to these petroclival chondrosarcomas [24]. These tumours tend to displace neurovascular elements laterally and superiorly [17]; however there are no previous reports of the relationship of the VC with the pICA in petroclival chondrosarcomas. A variable pICA displacement or destruction of the posterior vidian canal would limit the use of the VC as a reliable anatomical landmark.

The objective of this CT-based study was to evaluate the location of the pICA and its anterior genu relative to the posterior opening of the vidian canal in the presence of petroclival chondrosarcoma and to compare with the contralateral normal side.

## Methods

### Patients

Patients were selected for this retrospective single centre case–control study following local institutional approval (NS024) and without the requirement for informed consent.

A search of our institution’s electronic operative records was performed to identify consecutive petroclival chondrosarcoma patients (between 1/1/2001 and 31/12/2019). Patients were excluded if there was no definitive histological confirmation, tumour extension across the midline clivus, previous surgery to the petroclival region, or if thin section contrast-enhanced CT imaging did not adequately depict the pICA. Minimum imaging quality criteria comprised slice collimation < 0.7 mm and either pICA Hounsfield unit (HU) > 100 or pICA HU of 50–100 with contemporary MRA for correlation. Data collection included demographic and clinical information (age, gender, tumour characteristics). Contemporary MRI studies were reviewed to assess tumour dimensions and staging. Data on surgical complications and patient outcomes was obtained from the electronic patient record.

### CT acquisition, processing and analyses

The high-resolution CT studies through the temporal bones were performed (Siemens Healthcare, Erlangen, Germany) with scan parameters shown in Table 1.

**Table 1:** CT scan parameters

	IV contrast	IV contrast protocol	kV	auto mA	Noise index	Matrix	Collimation	Reconstructed slice thickness
Contrast-enhanced CT	Iohexol 300 mg iodine/ml (50 ml)	Acquired 3 min post injection	140	Range 100–515	2.8	512	Non-helical 4 × 5 mm detectors	0.625 mm with standard algorithm
CTA	Iohexol 300 mg iodine/ml (50 ml)	5 ml/s with a 50 ml/saline flush	80	Range 50–400	7	512	Helical 0.7 s rotation, pitch 0.984:1, slice interval 0.625 mm	0.625 mm with standard algorithm

A neuro-radiologist and a neurosurgeon analysed the CT studies by consensus. Petroclival chondrosarcoma and contralateral normal sides were analysed in a random order.

Multiplanar reformats were performed on a high-resolution PACS workstation (Sectra workstation, Sectra AB, Sweden). The images underwent standardised magnification and standardised window width. The pICA was defined as the segment extending from the external orifice of the carotid canal to the petro-lingual ligament [10]. The VC was defined as the canal located in the pterygoid process of the sphenoid bone, extending from just anterior to the foramen lacerum to the pterygopalatine fossa. The posterior opening of the VC was defined as where it started to splay at its dorsal margin. Any bony attenuation by the tumour at this site was documented, and, in such cases, the posterior opening of the VC was then estimated by reproducing the contralateral normal anatomy.

**Fig. 1:**
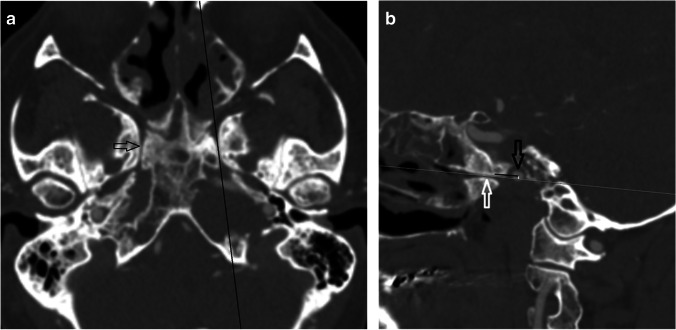
Patient 3. (**a**) Axial CT angiogram (CTA) on bone windows. Black line on the left demonstrates the sagittal oblique reformat plane for depiction of the relationship of the pICA with the VC. Open white arrow on the right indicates the VC. (**b**) Sagittal oblique reformat of the CTA on a normal contralateral side. The VC (open white arrow) and the pICA (open black arrow) are demonstrated with the trajectory of the canal indicated by the thin white line. The antero-posterior measurement is demonstrated as the distance from the mid-posterior opening of the VC (in the trajectory of the canal) to the mid AP point of the pICA cross-section (horizontal black line). The craniocaudal measurement is indicated as the vertical perpendicular distance from a line extrapolated posteriorly from the VC (in the trajectory of the canal) to the central point of the pICA cross-section (white vertical line). The line extrapolated from the posterior opening of the VC (in the trajectory of the canal) intersects the pICA (black open arrow)

**Fig. 2:**
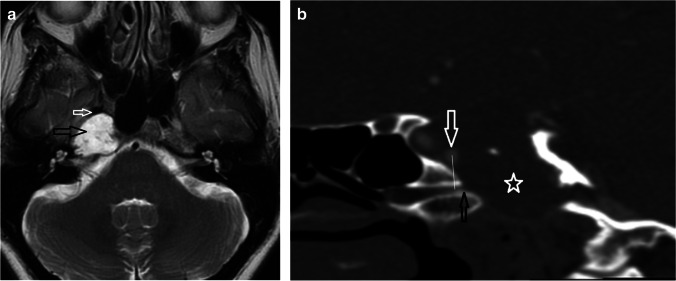
Patient 6. (**a**) T2w axial image demonstrating the hyperintense right petroclival chondrosarcoma (black open arrow) displacing the carotid artery flow void (white open arrow) anteriorly. (**b**) Sagittal oblique reformats of the CTA. In the presence of petroclival chondrosarcoma, the pICA (white open arrow) is displaced anteriorly and superiorly from the posterior opening of the VC (black open arrow). The lytic bony destruction of the chondrosarcoma is demonstrated (white star). The craniocaudal measurement is indicated as the vertical perpendicular distance (white vertical line) from the line of the VC to the central point of the pICA cross-section

Firstly, a standardised oblique sagittal reformat was performed in the line of the VC by aligning in the axial planes (Fig. 1) and the following was recorded (Table 2) (Figs. 1, 2):

1. Antero-posterior (AP) measurement between the VC and the pICA
2. Craniocaudal (CC) measurement between the VC and the pICA
3. Craniocaudal intersection.

**Table 2:** Description of the CT-based analyses of the VC and pICA anatomical relationship

Measurement	Description
Antero-posterior measurement between the VC and the pICA	AP distance (mm) from the mid-posterior opening of the VC (in the trajectory of the canal) to the mid AP point of the pICA cross-section (Figs. 1, 2). A positive figure was recorded if the pICA was posterior to the VC
Craniocaudal measurement between the VC and the pICA	Vertical perpendicular distance (mm) between a line extrapolated posteriorly from the mid-posterior opening of the VC (in the trajectory of the canal) to the central point of the pICA cross-section (Figs. 1, 2). A positive figure was recorded if the pICA was superior to this line
Craniocaudal intersection	Whether the line extrapolated from the posterior opening of the VC (in the trajectory of the canal) intersected the pICA cross-section or if it was superior/inferior to the pICA cross-section (Figs. 1, 2)
Coronal relationship between the anterior genu of the pICA and Vidian canal	The reformatted image was scrolled antero-posteriorly and the location of the anterior genu of the pICA was defined by its quadrant (inferomedial, inferolateral, superomedial, superolateral) relative to the VC (Fig. 3)

**Fig. 3:**
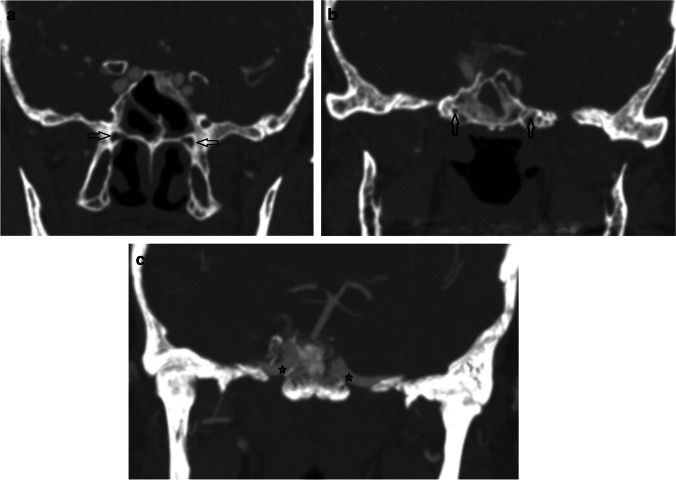
Patient 3. (**a**) Coronal reformat of the CTA perpendicular to the craniocaudal angle of the VC but without coronal obliquity extending from anteriorly to posteriorly (**a**–**c**). The location of the anterior genua of the pICAs (black stars) was recorded as superomedial relative to the VC (black open arrows) on either side

**Fig. 4:**
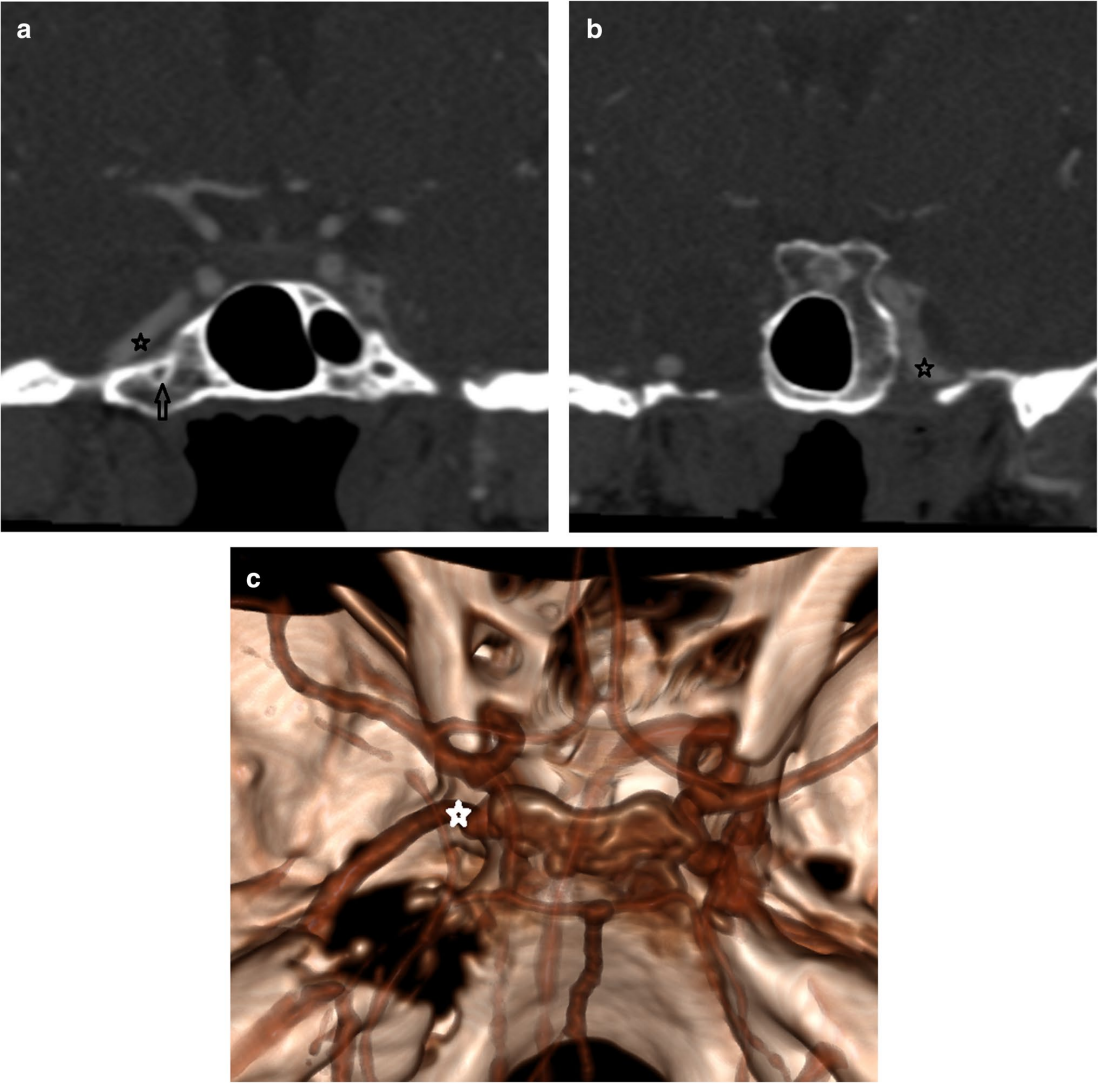
Patient 6. (**a**) Coronal reformat of the CTA perpendicular to the craniocaudal angle of the VC shows the displaced right anterior genu of the pICA (black star) to lie superolateral to the VC and demonstrating a shallow curve (black open arrow) on the chondrosarcoma side. (**b**) More posterior coronal reformat of the CTA shows the left anterior genu of the pICA (black star) to lie superomedial to the VC on the normal side with a better defined angle. (**c**) Volume-rendered image was used to locate the apex of the anterior genu (white star) when distorted

Secondly, a coronal reformat was performed perpendicular to the craniocaudal angle of the VC but without coronal obliquity. This was used to evaluate the direct coronal relationship between the anterior genu of the pICA and the posterior opening of the vidian canal (Table 2) (Fig. 3). The anterior genu corresponds to the junction of the petrous (C2) and lacerum (C3) segments as defined by Bouthillier et al. [1]. When the position of the anterior genu was not well defined on coronal reformats, it was correlated with volume-rendered imaging (Fig. 4).

Any narrowing of the pICA by the petroclival chondrosarcoma was documented as mild (30–50% area narrowing) or marked (greater than 50% area narrowing) and > 270^0^ contact of the chondrosarcoma with the ICA was recorded.

### Statistical analysis

Statistical analysis was performed using IBM SPSS (version 26) with a *p* value of < 0.05 being considered statistically significant.

Descriptive statistics summarised the patient demographics, previous surgery and tumour dimensions, Carlson staging and documented VC destruction, pICA narrowing by tumour, ICA contact with the tumour, craniocaudal intersection of the pICA in the trajectory of the VC and quadrant location of the anterior genu relative to the VC.

Continuous AP and CC measurements were compared between the chondrosarcoma and normal contralateral sides (paired *t* test if normally distributed and the Mann–Whitney test if not normally distributed).

The STROBE (Strengthening the Reporting of Observational Studies in Epidemiology) reporting guideline was implemented. Source data is available on request from the lead author.

## Results

### Patient, imaging and tumour descriptive characteristics

Twenty-two patients were eligible for study inclusion but 13 were excluded (1 primarily sellar chondrosarcoma and 12 cases that failed to meet image quality criteria). In total, the images of 9 patients (3 males, 6 females; median age 48.0, age range 17–74) were analysed in this study (Table 3).

**Table 3:** Patient and tumour characteristics

Patient no	Age/sex	Side	Axial dimension (mm)	Carlson grade	Regions involved*	Prior surgery**	VC erosion	ICA encased ***	ICA narrowing****
1	42 M	L	52 × 21	3	PS/EC/PF	RS	No	No	None
2	52 M	L	56 × 39	3	MCF/PS/EC/PF	None	Yes	Yes	Moderate
3	67F	L	30 × 23	3	MCF/PS/EC/PF	None	No	Yes	Mild
4	37F	L	25 × 22	2	PF	None	No	No	None
5	72F	R	33 × 24	3	MCF/PF	IT/RS	No	Yes	None
6	49F	R	27 × 19	2	MCF	None	No	No	Mild
7	17F	L	42 × 24	3	MCF/EC/PF	RS	No	No	Moderate
8	22 M	L	20 × 15	2	PS	None	No	No	None
9	74F	R	28 × 16	2	PF	None	No	No	Mild

^*^MCF middle cranial fossa, *PS* parasellar, *EC* extracranial, *PF* posterior fossa

^**^RS retro-sigmoid, *IT* infra-labyrinthine

^***^ > 270.^0^ ICA encasement

^****^Mild if 30–50% area narrowing and moderate if > 50% area narrowing

The long and short axial dimensions of the petroclival chondrosarcomas were 34.7 ± 12.5 mm and 22.6 ± 7.0 mm. Four tumours were Carlson stage 2 and 5 tumours were Carlson stage 3 [2]. There were 6 left-sided and 3-right sided lesions. There had been previous partial retro-sigmoid surgical resections in 2/9 patients and previous infra-labyrinthine and retro-sigmoid resections in one patient (Table 3). The presence of extra-osseous extension within posterior fossa, middle cranial fossa, parasellar and extracranial compartments is documented in Table 3.

There were 3/9 patients with mild pICA narrowing and 2/9 patients with moderate pICA narrowing by the chondrosarcoma. There was greater than 270 degrees ICA involvement in 3/9 patients (Table 3). The posterior opening of the VC was attenuated in one patient with a petroclival chondrosarcoma (Fig. 3). Data on the pre- and post-treatment patient Eastern Cooperative Oncology Group (ECOG) [20] performance status together with surgical complications and follow-up is tabulated (Table 4).

**Table 4:** Pre- and post-treatment performance status, surgical complications and outcomes

Patient no	Performance status pre-operatively (ECOG)	Surgical complication	Performance status at discharge (ECOG)	AdjuvantPBT	Length of follow-up (months)	Performance status post-treatment (ECOG)	Recurrence with further treatment
1	1	None	1	No	51	1	Yes/surgery
2	1	None	1	Yes	22	1	No
3	0	None	0	No	57	0	Yes/surgery
4	1	None	1	Yes	48	1	Yes/surgery
5	1	None	1	No	197	1	No
6	1	None	1	Yes	41	1	Yes/PBT
7	4	None	2	No	23	1/2	No
8	0	None	0	Yes	79	0	No
9	1	None	0	No	82	1	Yes/surgery

*ECOG* Eastern Cooperative Oncology Group

*PBT* proton beam therapy

### Vidian canal to pICA anatomical relationships on chondrosarcoma and contralateral control sides

The AP and CC measurements between the posterior opening of the VC and the pICA are shown in Table 4. The measurements were normally distributed as determined by the Shapiro Wilk test.

The AP measurement between the VC and the pICA was 3.9 ± 4.6 mm on the chondrosarcoma side and 7.2 ± 1.7 mm on the contralateral normal side. The pICA was located more anteriorly than the contralateral side in 8/9 of cases (Fig. 3) and more posteriorly in one case. The CC measurement between the VC and the pICA was 4.4 ± 3.7 mm on the chondrosarcoma side and 1.4 ± 0.8 mm on the contralateral side. The pICA was located more superiorly than the contralateral side in 6/9 of cases (Fig. 3), the same craniocaudal level in one case and more inferiorly in one case. The AP measurement differences were decreased on the chondrosarcoma side (*p* = 0.054) and CC measurement differences were increased on the chondrosarcoma side (*p* = 0.061) compared to the contralateral side.

The line extrapolated from the posterior opening of the VC in the trajectory of the canal less frequently intersected the pICA cross-section in the presence of chondrosarcoma (3/9 cases) than on the contralateral normal side (7/9 cases) (Table 5). The pICA cross-section was superior to this line in the remaining cases.

**Table 5:** VC to pICA anatomical relationships: comparison of chondrosarcoma with contralateral side

Patient no	AP (mm) pICA posterior to VC		CC (mm) pICA superior to VC		VC trajectory intersects with		pICA anterior genu quadrant relative to VC in coronal plane*	
	*Chondrosarcoma*	*Normal*	*Chondrosarcoma*	*Normal*	*Chondrosarcoma*	*Normal*	*Chondrosarcoma*	*Normal*
1	11.8	7	6.8	0.9	No	Yes	SL	SM
2	− 0.2 (anterior)	6.9	0.2	1.9	Yes	Yes	Centred on VC	SM
3	9.2	9.5	1.2	1	Yes	Yes	SM	SM
4	2.3	6.3	5.5	0.8	No	Yes	SM	SM
5	3.4	9.8	3.3	2.7	No	No	SM	SM
6	− 1.7 (anterior)	8	7.8	0.8	No	Yes	SL	SM
7	− 0.9 (anterior)	5.1	11.2	1.2	No	Yes	SL	SM
8	6.6	7.2	0.6	0.8	Yes	Yes	SM	SM
9	4.3	5.3	2.9	2.9	No	No	SM	SM

^*^SM superomedial, *SL* superolateral (SL)

**Fig. 5:**
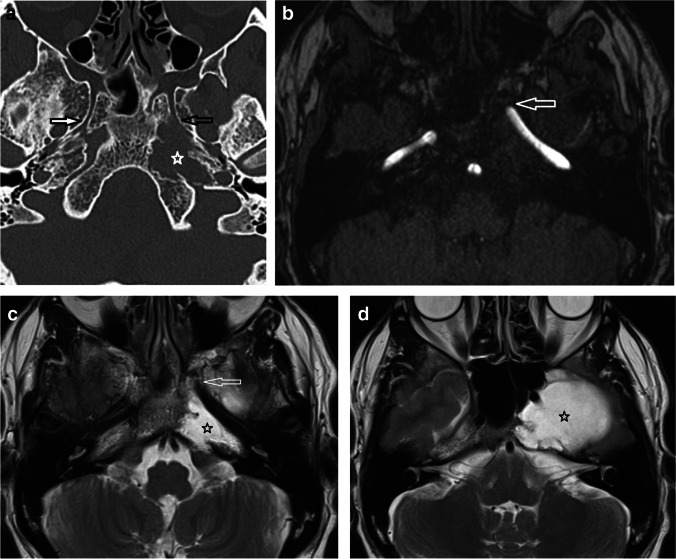
Patient 2. (**a**) Axial contrast-enhanced CT on bone windows demonstrates the destructive left petroclival chondrosarcoma (white star). The normal right sided VC (filled white arrow) is demonstrated with an intact and normally splayed posterior opening. The left VC has an eroded posterior opening (black open arrow). (**b**) Axial MRA and (**c**, **d**) T2w axial images confirm the location of the pICA (open white arrow) which is displaced in the line of the VC by the more posterior T2w hyperintense chondrosarcoma (black star)

Whilst the anterior genu was consistently located in the superomedial quadrant of all contralateral normal petroclival regions (Table 4), there was a more variable coronal location relative to the posterior opening of the VC in the presence of a chondrosarcoma (superomedial *n* = 5, superolateral *n* = 3, centred on the canal *n* = 1 (Fig. 5)).

## Discussion

**Fig. 6:**
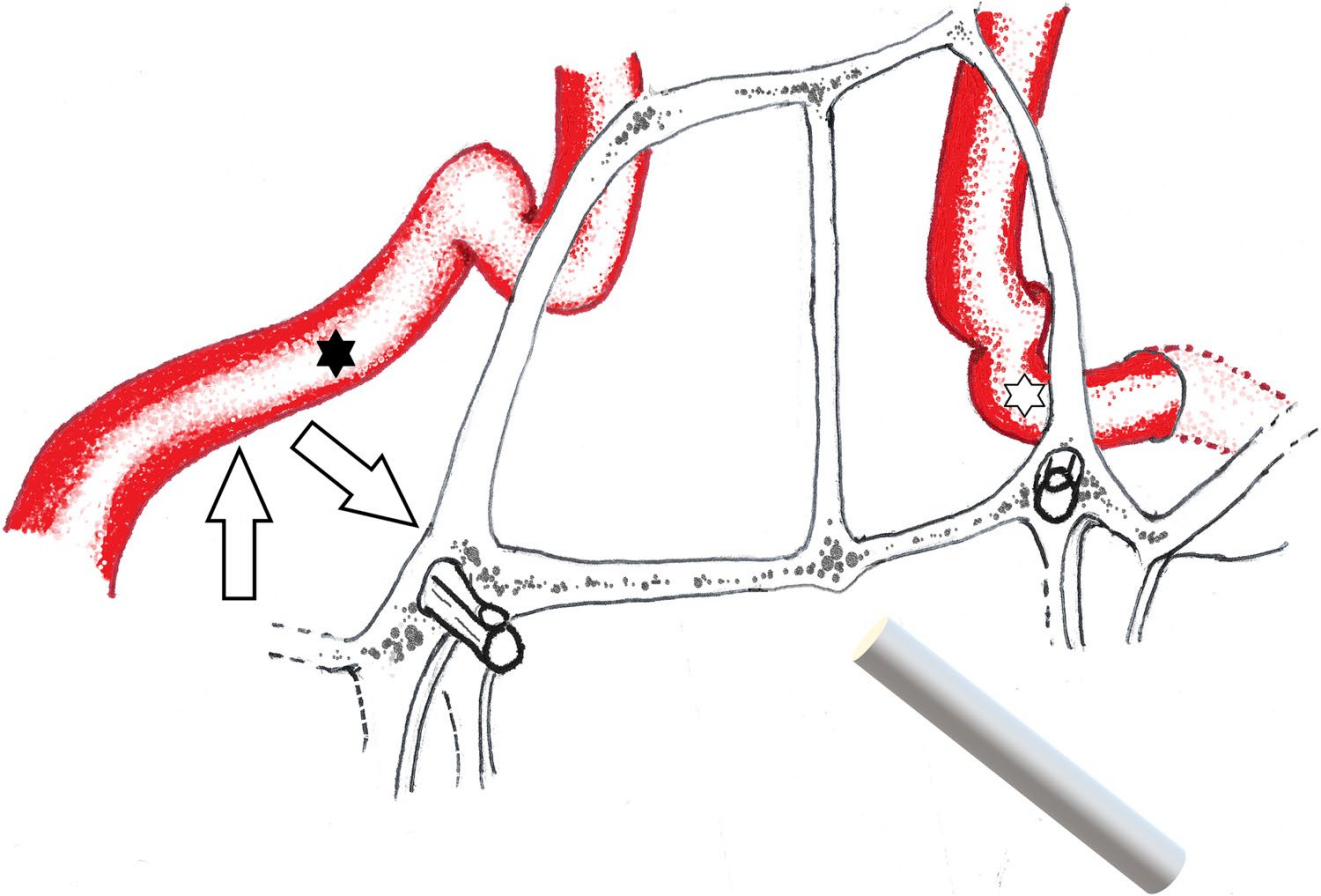
Diagrammatic demonstration of how chondrosarcomas most frequently displace the pICA. The right side represents the pattern of displacement in the context of a petroclival chondrosarcoma and is compared to the normal relationship of the pICA with the Vidian canal on the left. The pICA is more superiorly and anteriorly displaced relative to the posterior VC (arrows), whilst the anterior genu is seen to be superolateral (black star) rather than superomedial (white star) to the line of the VC. The antero-posterior plane is indicated by the line of the endoscope

This study indicates that the presence of a petroclival chondrosarcoma significantly and variably impacts on the anatomical relationship between the VC and the pICA. A petroclival chondrosarcoma most frequently displaces the pICA more superiorly and anteriorly relative to the posterior VC (Fig. 6). Whilst the pICA normally lies in the direct trajectory of the VC, a chondrosarcoma often displaces it superiorly relative to the line of the canal. In addition, the anterior genu of the artery pICA is expected to be superomedial to the dorsal VC; however a chondrosarcoma may displace it superolaterally. There may be rare occasions when the pICA is located directly in the line of the eroded posterior VC.

Injury to the pICA has a reported occurrence rate of 0.4–3.8% during traditional endoscopic endonasal approaches [13]. Endoscopic approaches to access the petroclival region in the coronal plane require fixed and consistent anatomical landmarks in order to locate the pICA [5–11, 14, 17]. The VC is the most established surgical marker to safely access the foramen lacerum [12, 23]. Drilling posteriorly along the medial and inferior aspect of the VC is described as a safe technique, since the posterior opening of the VC is reported to lie inferior to the anterior genu of the pICA. Alternative landmarks to localise the pICA have been evaluated such as the maxillary nerve [14], eustachian tube [22], vidian-eustachian junction [19], and the pterygo-clival ligament [18].

The presence of a petrous apex chondrosarcoma may potentially distort the pICA or erode the VC and impact on their expected spatial relationship. Whilst neuro-navigation may assist the surgeon during the surgical approach in the presence of distorted anatomy, movement of the pICA during the course of the dissection may lead to loss of accuracy. Whilst real-time intraoperative imaging such as Doppler ultrasonography [11] can help identify vital vascular structures, it is not a replacement for a clear understanding of the expected anatomical relationships along the coronal surgical trajectory. There have been few studies applying anatomical landmarks for the identification of the pICA and its anterior genu in the presence of central skull base tumours. Kassam et al.^9^ described the anatomical relationship in 25 surgical cases although the tumour characteristics were not described. Oakley et al. [19] successfully applied the vidian-eustachian junction to the location of the ICA in 9 patients with skull base tumours of which 3 were petroclival chondrosarcomas. Our results indicate that petroclival chondrosarcomas should be anticipated to displace the pICA superiorly from the trajectory of the VC, so maintaining its application as a safe landmark in almost all cases. However, in rare cases, the pICA may be displaced anteriorly in the line of the canal and so surgeons should be mindful of this potential hazard. The potential for destruction of the dorsal VC may also require the use of alternative anatomical landmarks to identify the pICA [18].

Whilst the main focus has been on cadaveric studies, these anatomical relationships have also been investigated on computed tomography in normal subjects [16, 23]. Our findings on the contralateral normal petroclival regions are consistent with those of Vescan et al. [23] who found the anterior genu of the pICA to be superomedial to the VC in all cases. Mason et al. [16] studied the vertical relationship of the pICA with the VC and demonstrated 34% of cases to terminate at the level of the canal when extrapolated posteriorly in the line of the palate. Whilst this conflicts with our finding of 7/9 cases intersecting the pICA in the craniocaudal axis, it should be noted that our study mimicked the surgical approach in the trajectory of the VC.

This study has limitations due to its retrospective design. In particular, the majority of eligible chondrosarcoma cases were excluded due to the absence of adequate contrast-enhanced thin section CT imaging. In addition, whilst stringent inclusion criteria for the depiction of the contrast-enhanced ICA were applied, formal CTA studies would be optimal for this analysis.

## Conclusion

Whilst the VC is normally a reliable indicator of the pICA location, conventional landmarks appear less reliable in the presence of petroclival chondrosarcoma. The pICA is usually superiorly displaced, and its anterior genu remains superior to the VC; however, rarely, the use of this anatomical landmark is precluded by anterior displacement of the pICA in the line of the VC. The inconsistent relationship between the VC and the pICA supports an individualised approach to determine anatomy of the VC and the pICA in the setting of a petroclival chondrosarcoma.
